# Knowledge on cervical cancer among Indian women in the state of Gujarat

**DOI:** 10.6026/97320630019999

**Published:** 2023-10-31

**Authors:** Nagooran Sivasubramanian, Mahalakshmi Beeman, Urviben Bharatbhai Patel, Vaghela Payalben, Ambiha R, Ekambaram Gnanadesigan

**Affiliations:** 1 Nootan College of Nursing, Sankalchand Patel University, Visnagar, Gujarat - 384315, India; 2 Department of Physiology, Nootan medical college and Research Centre, Sankalchand Patel University, Visnagar, Gujarat - 384315, India

**Keywords:** Awareness, knowledge, attitude, cervical cancer (cc), women

## Abstract

Women all around the world are affected by Cervical Cancer (CC). Nearly 5, 00,000 women were diagnosed with cervical cancer and more than 270,000 women die every year. Cervical Cancer is the second most typical form of Cancer in females worldwide. The aim of this study is to evaluate the effectiveness of an awareness programme on knowledge and attitude regarding CC among women. Quasi experimental research design was selected to evaluate the effectiveness of an awareness programme. Non-probability convenient sampling method was used to select 60 women from setting. Self-prepared multiple choice questionnaire was used to assess the knowledge level of women and 5 point likert scale (uni-dimensional scale for collecting opinion) was used to assess the attitude level of women. The Results were discussed by using mean, SD, paired "t" test and chi-square test. Data were collected & gathered. Knowledge levels were 2.87±1.50 and attitude level was 6.37±13.35 reported in the control group. But the knowledge and attitude levels in the experimental group were respectively 9.73±2.60 and 12.87±14.72. It concluded that the CC awareness program was successful in changing women's attitudes through knowledge.

## Background:

The most common gynecologic cancers in the world are still cervical cancer and ovarian Cancer. It is currently the 14thmost common Cancer overall and the 4thmost common Cancer in women worldwide [1]. Wide disparities in CC incidence and deaths were seen, with Sub-Saharan Africa having the highest incidence rates and developing countries accounting for 85% of all deaths [2]. 90% of Cervical Cancer deaths occur in low- and middle-income nations, where it is the 2nd most frequent malignancy among women worldwide. In India, there are roughly 96,922 new instances of cervical cancer detected each year [3]. World Health Organization has started a worldwide program to expand prevention, screening, and treatment efforts to eradicate CC as a public health issue in the 21st century [4]. In the United States, there were reportedly 2.96 lakhs women living with CC in 2020. Based on cases from 2016 to 2020, for every 100,000 women, 7.7 new cases of Cervical Cancer were diagnosed each year, and 2.2 women died from the disease annually [5]. Cervical cancer accounts for 6-29% of all malignancies in women in India. The state of Mizoram has the highest age-adjusted incidence rate of CC at 23.07/100,000, while the district of Dibrugarh has the lowest at 4.91/100,000 [6]. In low- and middle-income countries where there are neither structured screenings nor HPV vaccine programs, almost 90% of cervical cancer cases are discovered. Incidence and death of cervical cancer have more than halved in high-income countries since the introduction of systematic screening programs 30 years ago [7]. A delayed diagnosis and a poor prognosis for cervical cancer may result from a lack of knowledge of screening procedures, risk factors, and symptoms. Compared to urban regions, women in rural areas were more likely to be unaware of HPV infection (87.5%), cervical cancer (55%), its screening (75%), and the HPV vaccine (95%). There was relatively little knowledge of symptoms and risk factors in both rural and urban settings [8]. Therefore, it is of interest to document knowledge on cervical cancer among Indian women in the state of Gujarat.

## Methodology:

This study explains about, cervical cancer is the primary reason why women die and that women need to be educated about the disease. The researcher evaluates the impact of an awareness program on knowledge and attitudes about cervical cancer using a quantitative evaluative research approach. A Quasi-experimental before and after control group only research design was adopted and non-Probability convenient sampling technique was used to select 60 women. Thirty self-made questionnaires were created to assess the impact of an awareness program on women's knowledge about cervical cancer, with scores ranging from 0 to 30 representing inadequate, somewhat adequate, and adequate knowledge. A 5-point Likert scale has been used to evaluate their attitudes; Likert scale is one of the most fundamental and widely used psychometric tools in research in the social and behavioural sciences. Scores ranged from 0 to 120 were included and categorized as mentioned below.

0 = Undecided. 1 = Strongly Disagree,

2 = Disagree, 3 = Agree, and 4 = Strongly Agree.

These tools were used to collect data, which were then evaluated using inferential and descriptive statistics.

## Results:

**Figure 1:**
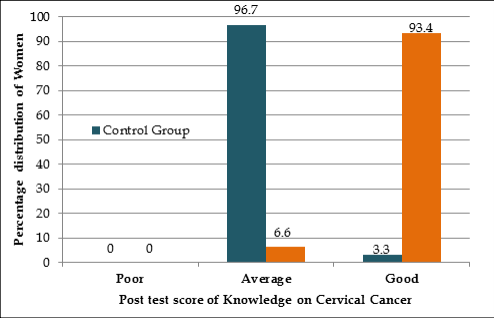
It shows the level of knowledge among women regarding CC after awareness program, in Experimental group 93.4% women having good knowledge, whereas in the control group 96.7% having average knowledge.

**Figure 2:**
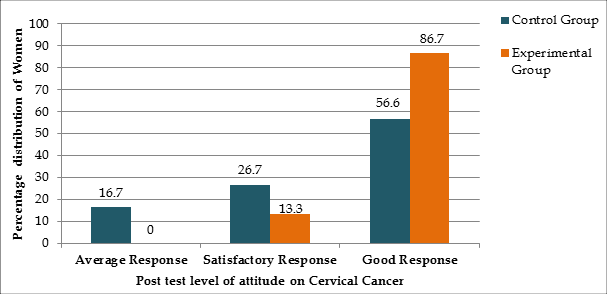
It shows that the post-test scores of attitude regarding Cervical Cancer; in Experimental group 86.74% women did good response, whereas in the control group 56.6.7% didgood response.

The age of women indicates that 9 (30%) are in the 20-30 years range, 14 (46.7%) are in the 31-40 years range, and 6 (23.3%) are in the 41-50 years range. According to the age of menarche, one (3.3%) is between the ages of 10 and 12, 26 (86.7%) are between the ages of 13 and 15, and 3 (10%) are older than 15 years. Only 2 (6.7%) women were married when they were between the ages of 13 and 15 and 16 and 18, the majority of women (63%) were over 21 when they got married, while 7 (23.3%) were between 18 and 21. In terms of the number of kids, around 16 (53.3%) women have two kids, 11 (36.7%) women have one kid, and just 3 (28%) women have three kids or more. According to an analysis of the respondents' women's yearly income, the majority 26 (86%) of them were below the poverty level of Rs. 50000, and only 4 (13.3% of them were over it. If any family members had a history of cancer, it was questioned of the women. 30 ladies (100%) said their family had not been impacted by cancer. The majority of women 20(66.7%) learned about cervical cancer from media sources like television and newspapers, 4 (13.3%) were learned from books and friends. It is noted that women 2(6.7%) of the remaining population learned about it from journals (Figure 1 Figure 2).

Table 1 show that the post test scores of awareness education on knowledge and attitude regarding CC among women in experimental and control group. In control group, knowledge level was 2.87±1.50 and attitude level was 6.37±13.35. However, in experimental group knowledge level was 9.73±2.60 and attitude level was 12.87±14.72. The Unpaired 't' test value of Knowledge was 12.52 and attitude was 10.79.It was high when measured against the value in the table (1.98). This demonstrates that there is a significant (at the 0.05 level) association between the post test score of the awareness program's effectiveness in terms of knowledge and attitude about cervical cancer.

## Discussion:

According to the results of the current study, awareness programs significantly improve women's attitudes and knowledge about CC. Another study by Archana (2019) found that there was a marked increase in knowledge of adolescent girls after exposing them to a structured teaching programme about cervical cancer. In comparison to their mean post-test knowledge score of 19.9 and the mean percentage of 82.91, the samples mean pre-test knowledge score was 11.18 with a mean percentage of 64.53. At the 0.05 level of significance, it was discovered that the mean post-test knowledge score was higher than the mean pre-test knowledge score (t = 24.916, P<0.01) [9]. According to another study, the participants' average age was 39.8±10.1 years. Although 82.9% of the participants said they had heard of cervical cancer, only 2.3% were aware that it could be discovered early and only 51% recognized that it could be prevented. More than 75% of the participants lacked sufficient understanding about cervical cancer. However, the vast majority of them (99.9%) were in favor of cervical cancer screening. Prior to the trial, none of them had undergone a cervical cancer screening. The survey respondents' general understanding about cervical cancer was low. To raise awareness and encourage these women to participate in cervical cancer screening, a sustained health education and screening program is required [10]. Another study conclusion indicated that the educational package on cervical cancer (EPCC) was helpful in increasing rural women's awareness of the disease because the women's mean post-test knowledge score on the disease was significantly higher than their mean pre-test score. Due to the high prevalence of the disease, there is an urgent need to educate women about cervical cancer prevention [11]. According to another study, the pre-test and post-test mean values were 7.41 and 23.2, respectively, and the SD of the values was 3.97 and 4.89.The SD was 15.79. The table value (1.67) was lower than the expected "t" value (25.58). It was evident that by using the proper interventions, sensitizing programs had a positive effect on women's understanding about CC screening [12, 13,14,15]. Daniel Adane Endalew carried out a community-based cross-sectional study from March 1 to March 30, 2019. A systematic sampling methodology was used to choose 268 respondents in total. The study included 260 respondents in all, with a 97% response rate. 26.2% of respondents had strong knowledge, and 3.8% had experience with cervical cancer screening. To promote awareness and use of cervical cancer screening through appropriate interventions, all interested parties must concentrate on women in the reproductive age group [16]. Another study carried out by Harshad Kumar in Karnataka, the majority of women (81.9% [68/83]) have limited understanding of CC and its screening (85.5% [71/83]). Out of 83 women, only 6 had received screening. Despite the fact that the ladies had previously seen doctors, neither they nor they were notified about the CC screening program [17].

## Conclusion:

It is concluded that the CC awareness program in the present study help improve women's attitudes and knowledge about cervical cancer. This study also highlighted the importance of raising awareness, enhancing knowledge and encouraging active research for health information and fostering experiences with informational sources for cervical cancer. Furthermore, the present study also suggests developing health extension programs with appropriate interventions in order to encourage women's screening behaviour across all populations.

## Financial support and sponsorship:

Nil

## Figures and Tables

**Table 1:** Mean, SD and 't' test value of knowledge and attitude ratings of Experimental and Control group following cervical cancer awareness program.

**S.No**	**Variables**	**Post Test**		**Unpaired "t"test**	**Table Value**	**Level of Significance at 0.05 level**
		Mean	SD			
Level of Knowledge						
1	Control Group	2.87	1.5	12.52	1.98	Significant
2	Experimental Group	9.73	2.6			
Level of Attitude						
1	Control Group	6.37	13.35	10.79	1.98	Significant
2	Experimental Group	12.87	14.72			
